# Dr. Wm. H. Gregg

**Published:** 1915-08

**Authors:** 


**Dr. Wm. H. Gregg** (not listed in Polk), P. & S. 1857, died in

St. Cloud, Fla., June 30, aged 85. A native of Elmira, he served as interne in the Charity Hospital on Blackwell's Island, practiced for a time in N. Y. City and later in Port Chester, N. Y. He was surgeon of the 69th N. Y. Volunteers and later Hospital Surgeon of the 8th Brigade and Senior Inspecting Surgeon, during the Civil War. He was prominent as a chemist, mineralogist and ornithologist.

